# PP85 Access To Treatment For Hearing Loss In Chile: Do All People Have The Same Opportunities? A Scoping Review

**DOI:** 10.1017/S0266462325103024

**Published:** 2025-12-29

**Authors:** Erica Aranha Suzumura, Francesca Scandurra, Christina Schwarz, Michael Urban

## Abstract

**Introduction:**

Chile has been conducting various efforts toward reforming the healthcare system, yet significant discrepancies persist in the equitable access to and utilization of healthcare resources. The aim of this scoping review was to map the existing literature on hearing health care and treatments for hearing loss (HL) in Chile to identify local determinants that may contribute to stratification of access to hearing health care.

**Methods:**

The Joanna Briggs Institute guidance for scoping reviews was followed. The PCC criteria (Population, Concept, Context) was used to guide development of the search strategy. Searches were conducted in MEDLINE (PubMed), the Cochrane Library, and Science Direct databases and supplemented by a manual search. Searches were limited to publications from 2000 to June 2023, with no restrictions on language or publication type. Two independent reviewers screened all retrieved references, assessed the eligibility following the PCC criteria, and charted data of the eligible publications. Disagreements were solved through discussion with a third reviewer. A structured narrative synthesis of findings was conducted.

**Results:**

The search yielded 506 unique records for screening, of which 104 full-text publications were assessed and 39 were included in the review. The evidence revealed that the treatment of HL is publicly financed for children younger than four years (any type of HL, degree moderate or higher), the elderly aged 65 years or older (bilateral HL requiring a hearing aid) and people at least four years of age diagnosed with total deafness (cochlear implant candidates). However, for individuals aged between four and 64 years who have HL but are not totally deaf, there is not a clear rehabilitation pathway or dedicated public reimbursement.

**Conclusions:**

Age and degree of HL might be the most prominent determinants of access to hearing health care in Chile. Diagnosis and treatment of people aged four to 64 years with moderate to severe HL might incur out-of-pocket expenses or even have no access to care for HL. These findings highlight the need for public health policies to promote equitable access to hearing health care in Chile.

